# A Middleware Solution for Wireless IoT Applications in Sparse Smart Cities

**DOI:** 10.3390/s17112525

**Published:** 2017-11-03

**Authors:** Paolo Bellavista, Carlo Giannelli, Stefano Lanzone, Giulio Riberto, Cesare Stefanelli, Mauro Tortonesi

**Affiliations:** 1 DISI—Department of Computer Science and Engineering, University of Bologna, Bologna 40136, Italy; paolo.bellavista@unibo.it (P.B.); stefano.lanzone@studio.unibo.it (S.L.); 2 DMI—Department of Mathematics and Computer Science, University of Ferrara, Ferrara 44122, Italy; 3 DE—Engineering Department, University of Ferrara, Ferrara 44122, Italy; giulio.riberto@unife.it (G.R.); cesare.stefanelli@unife.it (C.S.); mauro.tortonesi@unife.it (M.T.)

**Keywords:** Internet-of-Things, smart city, spontaneous connectivity, opportunistic networking, distributed actuation

## Abstract

The spread of off-the-shelf mobile devices equipped with multiple wireless interfaces together with sophisticated sensors is paving the way to novel wireless Internet of Things (IoT) environments, characterized by multi-hop infrastructure-less wireless networks where devices carried by users act as sensors/actuators as well as network nodes. In particular, the paper presents Real Ad-hoc Multi-hop Peer-to peer-Wireless IoT Application (RAMP-WIA), a novel solution that facilitates the development, deployment, and management of applications in sparse Smart City environments, characterized by users willing to collaborate by allowing new applications to be deployed on their smartphones to remotely monitor and control fixed/mobile devices. RAMP-WIA allows users to dynamically configure single-hop wireless links, to manage opportunistically multi-hop packet dispatching considering that the network topology (together with the availability of sensors and actuators) may abruptly change, to actuate reliably sensor nodes specifically considering that only part of them could be actually reachable in a timely manner, and to upgrade dynamically the nodes through over-the-air distribution of new software components. The paper also reports the performance of RAMP-WIA on simple but realistic cases of small-scale deployment scenarios with off-the-shelf Android smartphones and Raspberry Pi devices; these results show not only the feasibility and soundness of the proposed approach, but also the efficiency of the middleware implemented when deployed on real testbeds.

## 1. Introduction

Today, it is estimated that some 15 billion devices are connected, and this number is set to explode to 50 billion by 2020, particularly in and around urban centers [1]. These devices are often organized in systems or networks of advanced sensors, which capture and disseminate multimedia data sets, often of considerably large size. In addition, a steadily growing selection of devices (not only smartphones, tablets, and laptops, but also wearable devices, smart TVs, smart electric appliances, and any kind of gadget ready for the Internet of Things) equipped with multiple network interfaces (LTE/4G, WiMax, WiFi, Bluetooth, Ethernet, etc.) and sophisticated sensing capabilities are entering the market, thus increasing even further the amount of sensing data generated. For instance, on top of at least two cameras and a microphone, modern smartphones feature an iris scanner, a pressure sensor, a fingerprint reader, an accelerometer, a gyroscope, a barometer, a proximity sensor, a compass, a heart rate sensor, a light sensor, and a magnetometer.

In particular, Smart Cities worldwide are embracing Internet of Things (IoT) technologies to streamline their operations and meet the growing expectations of their citizens. Today, citizens in the most vibrant cities are already seeing many initiatives designed to make urban services smarter, whether for transportation, parking, lighting, traffic and waste management, safety, or law enforcement. Urban services powered by the Internet will certainly enhance citizen quality of life, but developing this new generation of services requires to address challenging aspects, such as efficient packet dispatching in greatly dynamic and possibly partitioned wireless networks.

The paper has the specific goal of supporting Wireless IoT Applications in sparse Smart City environments by considering the many issues that the target scenario raises at different and heterogeneous levels, ranging from single- and multi-hop communication and inter-node coordination (by also considering dynamic network partitioning), to the management of distributed applications’ remote deployment and upgrades. These applications are expected to implement smart information dissemination functions on top of a distributed architecture of software components running on top of fixed sensor systems, sparse mobile nodes nomadically roaming and interacting with one another opportunistically, edge devices located in proximity of either raw data sources or information consumers, and in the Cloud via Internet-enabled mobile devices sharing their connectivity to the Web. 

The raw (sensing) data collection and processing functions implemented by Wireless IoT Applications are significantly more complex than those usually found in Wireless Sensor Network (WSN) applications. In fact, WSNs are usually designed and deployed to achieve very specific goals that are well-defined at service implementation time, and are thus unlikely to be exploited for different goals, either at the same time or in different time periods. For this reason, nodes of WSNs are usually configured in a specialized fashion, with limited requirements to drastically modify their behavior (even if configurability and upgradeability of software components may be considered as a plus). Instead, Wireless IoT Applications in sparse Smart Cities have a much more heterogeneous, dynamic, and general-purpose nature; they typically implement many different but concurrent data collection and processing tasks, and provide a set of services that can significantly change over their lifetime. 

We envision that smartphones will play a key enabling role in Wireless IoT Applications, as they will be opportunistically adopted to dynamically extend and enhance Smart City environments, by dispatching data as well as by behaving as IoT devices that can monitor/control the surrounding environment. In particular, by taking advantage of their pervasive availability and increased software/hardware capabilities, smartphones will become an integrated part of sparse Smart Cities and will collaborate to support multi-hop connectivity, by dynamically and directly interconnecting one another (i) to create single-hop links in a peer-to-peer fashion and (ii) to collaboratively dispatch packets by acting as intermediary nodes between senders and receivers (in addition to exploiting the “more traditional” but sporadic availability of infrastructure connectivity, e.g., based on IEEE 802.11 Access Points). 

To facilitate a full understanding of the proposal, the manuscript also illustrates how our solution enables and supports the implementation of a sample Wireless IoT Application, characterized by a pool of Raspberry Pi devices acting as sensors periodically gathering raw data (pictures via webcams), processing them (by comparing current and previous pictures), and sending alert messages if given conditions apply (the two pictures differ more than a threshold). Internet-enabled Android smartphones, widely available in the envisioned sparse Smart City scenarios, act as controllers, located in the proximity of sensor nodes themselves and dispatching information (e.g., alert messages) and commands (e.g., configuration parameters to modify the image comparison threshold) as well as novel software components (e.g., containing a different algorithm for image comparison) between a (logically) centralized cloud application and sensor nodes themselves. 

The paper takes advantage of our previous experiences in the spontaneous networking research field [2,3] by originally providing novel solutions to support Wireless IoT Applications in sparse Smart City scenarios that consider the many issues that developing and deploying applications in sparse Smart Cities raise. More specifically, the proposed solution provides effective mechanisms to manage single subnets, to dispatch packets at multi-hop distances between subnets managed in an uncoordinated way, to detect packet dispatching failures by also taking proper countermeasures based on store-and-forward policies, to coordinate/manage controller and sensor nodes by specifically taking into consideration that the whole network could become partitioned at provisioning time, and also to make easier the development/deployment of novel services by providing general-purpose controller/sensor software components as the basis of new and specialized applications. In particular, the paper presents the Real Ad-hoc Multi-hop Peer-to peer-Wireless IoT Application (RAMP-WIA) middleware, which extends our previous solution along 4 primary guidelines: (i) proper management of single-hop wireless link among sensors, controllers, and other nodes of the network to increase the dynamicity of the overall network (Section 4); (ii) effective multi-hop packet management also considering that sparse networks are likely to be partitioned and thus packets dispatched among sensors and controllers should be properly managed by intermediary nodes (Section 5); (iii) reliable and resilient actuation of sensor nodes specifically considering that controllers could be interested in issuing commands only if (most of) sensor nodes are actually reachable, otherwise the command should be safely aborted (Section 6); and (iv) dynamic upgrade of sensor nodes by supporting the distribution and deployment of new software components on-the-fly (Section 7).

## 2. Related Work

Smart City environments and IoT applications have received a considerable attention from scientific literature in recent years. Just to provide some notable examples, consider that Smart Cities can provide a new generation of real-time and time-critical, location-, social-, and context-aware services to their digital citizens, such as for emergency and health-care [4], surveillance [5], entertainment, and social good [6,7]. Recent research activity has been focused on many different IoT-related topics such as event forecasting [8], WSN routing protocols [9], multi-sensor information fusion [10], business model and profit maximization [11], ontologies [12], service models [13], quality of experience [14], and even advanced concepts for the prioritization of raw data processing and information dissemination such as Quality of Information (QoI) [15] and Value-of-Information [16]. Researchers have also developed a multitude of application-specific solutions for issues in diagnostics [17,18], environmental monitoring [19,20], and social interest [21]. 

However, designing and developing Wireless IoT Applications for sparse Smart City environments still represents a significant challenge which calls for the development of new models and paradigms at both the architectural and the communication paradigm level. From an architectural point of view, Fog Computing represents one of the most interesting and relevant research areas supporting the development and management of Wireless IoT Applications in Smart City environments. For instance, Stack4Things is a Fog Computing platform for IoT applications proposing a Cyber-Physical System with Functions Virtualization (CPSFV) to manage sensors and actuators, group them together, make easier their mutual interaction, and enable the specialization of their behavior [22]. The last task is performed via contextualization, defined as the Cloud-controlled injection of code, in the form of plugins, into any sensor/actuator managed by the Stack4Things platform. Manzalini and Crespi [23] proposed the Edge Operating System (EOS), a software architecture that leverages concepts and tools from Software Defined Networking (SDN) and Network Functions Virtualization (NFV) to exploit the processing power of network infrastructure elements. 

Information Centric Networking (ICN) is another promising research field presenting solutions to support application communication in wireless and heterogeneous networking environments, also allowing easier deployment and management of Wireless IoT Applications in sparse Smart Cities. For instance, Amadeo et al. presented an ICN architecture for IEEE 802.11 MANETs called CHANET [24]. CHANET relies on naming to identify the content and it makes use of broadcast for the transmission of both interest packets and data. Other interesting techniques implemented in CHANET to increase effectiveness in the wireless environment are the overhearing of nearby nodes’ transmissions and the local decision-making processes regarding packet forwarding. Mendes [25] described an opportunistic, content-centric architecture that takes advantage of the increasing number of pervasive systems available today to share content. The proposed Information and Context Oriented Networking (ICON) framework encompasses techniques that come from both the research fields of data-centric networking and opportunistic networking. ICON exploits caching strategies developed in ICN to share and place contents across devices in the network, and it relies on opportunistic strategies based on social- and location-based information, and the knowledge about application data usage, to route and forward the content to nodes interested in it. Finally, Delmastro et al. [26] proposed mobile social networks that are specifically designed for Smart Cities with the goal of pushing citizens to actively participate in generating and sharing information related to quality of life. 

How to flexibly and dynamically manage packet dispatching in IoT environments is an important topic at the communication level. For instance, Network Coding and Power Control based Routing (NCPCR) is a solution aimed at saving energy when dispatching packets on top of unreliable wireless networks [27]. In particular, NCPCR exploited network coding and was based on dynamic management of the transmit power. Pin-Yu Chen et al. [28] proposed the adoption of cognitive radio in IoT environments to access the spectrum in a dynamic and efficient fashion, in particular by proposing a hybrid interference-aware flooding scheme to improve information delivery among nodes. Al-Turjman [29] presented and compared several techniques for the IoT paradigm, differing in relation to energy consumption, cost, and delay. In particular, the newly proposed Multipath Disruption-Tolerant Approach (MDTA) routing enabled minimization of message delivery delay while reducing the exploitation of roadside fixed nodes in vehicular networks with sensor capabilities. Finally, Efficient IoT Communications based on Ant System (EICAntS) allowed improvements of network performance in terms of node lifetime and energy consumption by adopting an ant-inspired routing protocol in IoT environments [30]. 

At the coordination level, Wireless IoT Applications can benefit from management decisions based on dynamically-gathered context information. For instance, Pilloni et al. [31] proposed a consensus approach aimed at maximizing the lifetime of groups of nodes, with the goal of fulfilling QoI requirements. To this purpose, nodes coordinate one each other via a consensus approach specifically considering the lifetime of nodes, storage capacity, processor capabilities, and available bandwidth to optimize the allocation of tasks. Cao et al. [32] more specifically presented state-of-the-art literature proposing coordination solutions in the industry sector. In particular, it presents several work aiming at achieving consensus among several agents in distributed scenarios. Caraguay et al. [33] proposed a solution to improve network management also considering novel networking paradigms such as SDN and NFV. In particular, the proposed SELFNET solution allows to easily query large amount of monitored data, gathered from heterogeneous sources. In addition, information provided by SELFENT can be correlated to provide enhanced information and improve the network Quality of Service (QoS) via proper coordination and enforcement of proper actions. 

While state-of-the-art solutions provide interesting insights in specific issues of multi-hop wireless networks, let us note that they deal with the many aspects of their target scenario in a silos-based fashion, thus failing to provide a complete and architectural solution covering topics at different layers. For instance, while Delmastro et al. [26] targeted the collaboration of citizens in Smart Cities also based on opportunistic sensing and communication, their study provides details only about the developed application; moreover, it reports performance results mainly related to the users’ quality of experience and to simple subnets (with no support for inter-subnet routing). Instead, Sotres et al. [34] mainly focused on the many practical aspects related to the deployment of a large-scale Smart City infrastructure, while neglecting issues related to the development and deployment of novel applications on top of the infrastructure itself. We believe that the development of comprehensive and general-purpose methodologies (and the prototyping of the associated tools) to support the development of Wireless IoT Applications at the communication, coordination, and management levels has been relatively neglected so far. With these motivations, the rest of the paper presents a solution supporting the easy development and management of monitoring and control applications in sparse Smart Cities on-top-of multi-hop infrastructure-less wireless networks. Our proposal provides researchers and practitioners in the field with solutions ranging from the communication layer (by supporting mechanisms for single subnet configuration as well as opportunistic multi-hop packet dispatching), to the coordination layer (by ensuring coordination among nodes participating to the same Wireless IoT application), till the remote application management (by making easier the remote deployment and upgrade of distributed applications in sparse Smart City environments).

## 3. The RAMP Middleware for Wireless IoT Applications

**Figure 1:**
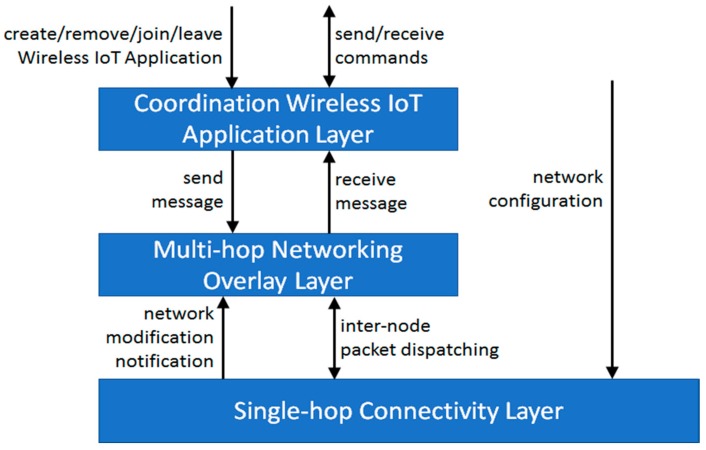
High-level overview of the three-layer reference architecture.

We have identified three primary features that should be supported to foster the development of Wireless IoT Applications in sparse Smart Cities: single-hop connectivity management to make easier the dynamic creation of device-to-device connections, opportunistic multi-hop packet management to properly dispatch packets at multi-hop distance also in case of temporarily partitioned wireless networks, and application coordination tools to facilitate the dissemination and deployment of Wireless IoT Applications. The rest of the section presents a three-layer reference architecture supporting the required features (see Figure 1) and outlines the main functionalities that each layer should support. This architecture is implemented in our novel RAMP-WIA solution (based on and enhancing our RAMP middleware), which is described in the following sections and which provides novel tools addressing specific issues of monitoring and controlling Wireless IoT Applications in sparse Smart City environments.

Starting from the bottom layer of the proposed reference architecture, the main objective of the Single-Hop Connectivity Layer is to support the creation and access of IP subnets based on wireless protocols widely available on off-the-shelf mobile nodes. In particular, the layer should provide the capability of:

- manually configuring IP connectivity. In this case, developers can optimize networks to achieve specific goals based on the full knowledge of the environment, that is, node location and movements. However, since nodes may have unpredictable movement patterns, it may be unfeasible (or very complex with high costs in terms of information dissemination and centralized control) to manually configure each and every network interface of nodes;
- managing IP connectivity in a completely autonomous manner. The layer should be able to modify network configurations to take advantage of possibly available nodes in the same location, both accessing and providing connectivity in a peer-to-peer-fashion. In this manner, it is possible to achieve a high degree of dynamicity while completely hiding any link connectivity complexity.

In both cases, the layer should provide easy to use Application Programming Interface (API) to: (i) create new IP subnets and access to already available ones, by hiding (whenever possible) the complexity of managing heterogeneous wireless protocols; (ii) finely tune the degree of dynamicity that the network should have in case of automatic configuration; and (iii) notify link connectivity modification to efficiently make other components aware of new connectivity opportunities. Practical examples of how a middleware implementation of this layer can work, and with which advantages, are reported in Section 4; in particular, our Link Connectivity Component (LCC) (inspired by WiFi-Opp [35]) implements this layer for both IEEE 802.11 and Bluetooth in manual as well as automatic manner, on top of off-the-shelf Android devices.

The goal of the Multi-hop Networking Overlay Layer is to support node by node packet delivery among nodes via multiple single-hop links provided by the layer below. To this purpose, we identify two primary features this layer should support:

- it should allow to easily exploit IP subnets provided by the Single-hop Connectivity Layer by dispatching packets among nodes in a transparent manner, with no need of manually configuring the operating system routing tables to correctly deliver information to the receiver. To this purpose, we exploit the RAMP middleware which supports multi-hop collaborative communications independently from how underlying (possibly heterogeneous) links/IP sub-networks have been created. RAMP nodes cooperate at the middleware layer to dispatch packets, with no need to modify routing tables at the operating-system level, thus achieving the degree of dynamicity needed in sparse Smart City scenarios [2,3,36];
- it should efficiently manage network failures and partitioning that have to be considered as a frequent event in the sparse Smart City scenarios. To this purpose, we have significantly extended our RAMP middleware by designing and implementing the RAMP Opportunistic Networking (RON) component, transparently supporting the efficient management of packets in case of the destination node is temporarily unreachable. In case of packet dispatching failure, RON persists packets and then automatically retries to transmit them to the destination in case of link connectivity modifications (even duplicating packets to maximize packet delivery probabilities), that is, since a node moved from one location to another providing access to new IP subnets or the Single-hop Connectivity Layer has modified the configuration of a network interface.

API at the Multi-hop Networking Overlay Layer can allow to tune the behavior of nodes to achieve a proper tradeoff among resource sharing to support packet management and resource preservation to avoid too much overhead (additional details on our implementation in Section 5). 

Finally, the Coordination Wireless IoT Application Layer provides tools to simplify the development of sensor and controller nodes. This layer hides the complexity of node coordination, such as by enabling the transparent dispatching of commands and updated software components (additional insights in Section 6 and Section 7 respectively) among sensors and coordinators related to the same Wireless IoT Application. At the same time, it supports the tuning of packet management to achieve a proper tradeoff among packet delivery probabilities and cost of packet management. For instance, when sending a message, sensor nodes can specify a proper expiry value after which messages should be discarded even if not yet correctly delivered to the destination by the Multi-hop Networking Overlay Layer. From an architectural point of view, it exploits primitives provided by the layer below to support the dispatching of messages with end-to-end visibility by adopting four different semantics:

○ one-to-one, to send packets from a sender to a given receiver, for example, to send a document via a file sharing application;
○ one-to-any, to send packets from a sender to any node of a set of receivers, for example, to send sensed data to one of the available sinks in a sparse Smart City environment;
○ one-to-many, to send packets from a sender to every node of a set of receivers, for example, to send a message to a group of a chat application;
○ one-to-all, to send a packet to every node of the network, for example, an alert message in case of fire hazard.

## 4. The Link Connectivity Component

To develop and deploy Wireless IoT Applications actually fitting the sparse Smart City environment, it is required to thoroughly and realistically consider not only the dynamicity of nodes carried by users, but also the actual availability of wireless interfaces in off-the shelf devices. 

On the one hand, in Smart Cities characterized by sparse nodes collaborating with one another to support Wireless IoT Applications, the dynamicity of the network mainly depends on the mobility of users; by moving from a location to another they access different IP subnets, increasing the probability of spreading packets towards their destinations. However, if users (and thus their smartphones) do not move for a relatively long time period, packet spreading is not possible, since smartphones keep exploiting the same IP subnets. 

On the other hand, to provide a solution that can be realistically adopted, requirements and constraints stemming from real-world environments must be taken into proper consideration. In particular, since nodes composing the overlay network are mainly based on off-the-shelf devices such as smartphones and laptops, device-to-device communication can be based on either IEEE 802.11 or Bluetooth, the only wireless medium-range protocols that are currently widely available. A main consequence of adopting IEEE 802.11 and Bluetooth is that each device can typically perform one-hop connections; IEEE 802.11s meshes and Bluetooth scatternets are not easily available on off-the-shelf devices. In other words, real-world scenarios do not allow typical WSN deployments where packets flow from node to node exploiting a single wireless interface in a multi-hop fashion as is possible by employing technology such as ZigBee. In fact, IP subnets provided by smartphones communicating in a device-to-device fashion may represent many connectivity islands of a statically partitioned network. Even the adoption of a multi-hop overlay layer based on opportunistic networking (with its capability of persisting and dispatching packets again) may be inadequate to correctly deliver packets, since the limited dynamicity of networks can prevent to disseminate packets towards their destination. 

In sparse Smart City scenarios, the probability of successfully dispatching packets to destination can be maximized by dynamically modifying the attachment network to increase the number of nodes where packets are transmitted to. To this purpose (and inspired by WiFi-Opp [35]), we have designed and developed the Link Connectivity Component (LCC) that allows re-configuring IP subnets by: (i) dynamically accessing different hotspots; and (ii) dynamically switching the role of smartphones from client to hotspot (and vice versa). It is worth noting that the overall network is still partitioned, since the adoption of IEEE 802.11 and Bluetooth does not allow to create multi-hop wireless links. However, the increased dynamicity of the connected networks allows to spread packets to a larger set of nodes, by taking advantage of the different network opportunities and IP subnets that may become available.

**Figure 2:**
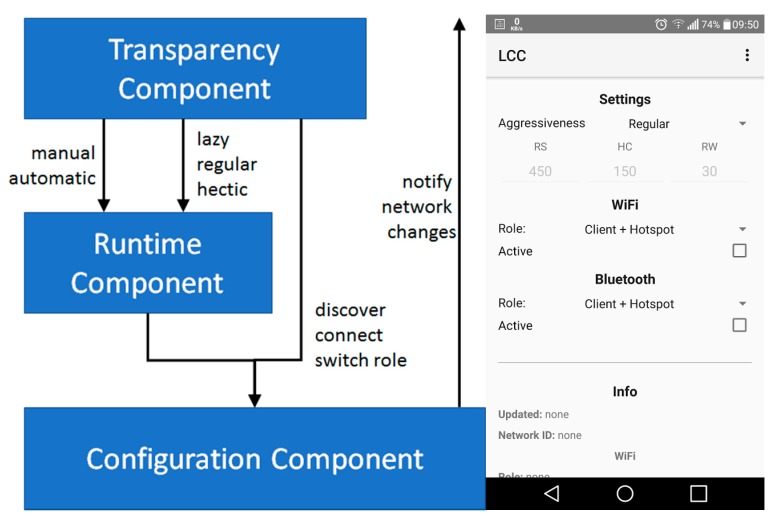
Link Connectivity Component (LCC) architecture (**left**) and Android GUI (**right**).

Delving into finer details, LCC is based on three layered sub-components (see Figure 2, left):

- the Configuration Component (CC) layer provides an abstract access to networking devices, hiding the complexity of accessing heterogeneous wireless protocols such as IEEE 802.11 and Bluetooth. In particular, it provides API to discover available hotspots, to connect to a given peer, and to switch the role from client to hotspot and vice versa. In addition, it provides a listener interface to allow external components to be notified about any network configuration modification in an event-based fashion, that is, to notify state change from client to hotspot or the modification of the hotspot a client is attached to;
- the Runtime Component (RC) exploits CC to dynamically modify network interface configurations, by periodically modifying the accessed hotspots and switching between client and hotspot roles. In particular, RC exploits two periods, that is, Hotspot Change (HC) period and Role Switch (RS) period, to specify how long a network interface should exploit the same hotspot and change the client/hotspot role respectively. In addition, in case a network interface has the client role but there is not any hotspot available, it switches to the hotspot role after a random period in the [RW/2, RW] range based on the Random Wait (RW) parameter. Note that randomness is exploited to avoid that multiple clients in the same location switch to the hotspot role simultaneously;
- the Transparency Component (TC) provides users with an easy-to-use Graphical User Interface (GUI) to specify if LCC should modify network configurations either manually or dynamically. In the former case, it allows users to configure the interfaces by directly interacting with CC. In the latter case, it provides the capability of specifying the policy RC should adopt.

Based on the three RC periods, we have developed the following pre-defined policies users can activate from TC:

- lazy, with HC = 300 s, RS 900 s, RW = 45 s;
- regular, with HC = 150 s, RS 450 s, RW = 30 s;
- hectic, with HC = 30 s, RS 90 s, RW = 15 s.

Note that the lazy, regular, and hectic policies provide increasing degrees of dynamicity, allowing different tradeoffs between effectiveness and overhead. In fact, the more frequently RC modifies network configurations, the more probable (and more quickly) packets are dispatched towards their destination. However, network dynamicity has a cost in terms of increased network configuration instability, that could negatively affect active communications, such as by abruptly interrupting active inter-node packet dispatching procedures, thus requiring proper retransmissions.

Finally, it is worth noting that the current implementation of the CC layer exploits the Android API (Figure 2, right shows a GUI screenshot) for IEEE 802.11 and Bluetooth (not exploiting the specific features and APIs of WiFi Direct and Bluetooth Low Energy to ensure backward compatibility). However, the overall LCC architecture is extendible to easily add CC implementations for other operating systems and wireless interfaces, as long as they provide API to discover/access hotspots and switch among client/hotspot roles. Moreover, the adoption of different CC implementations does not affect RC and TC layers and thus their adoption can be regarded as general-purpose and cross-platform. The source code of LCC can be found at https://github.com/DSG-UniFE/lcc.

## 5. The RON Component for Networking Overlay

**Figure 3:**
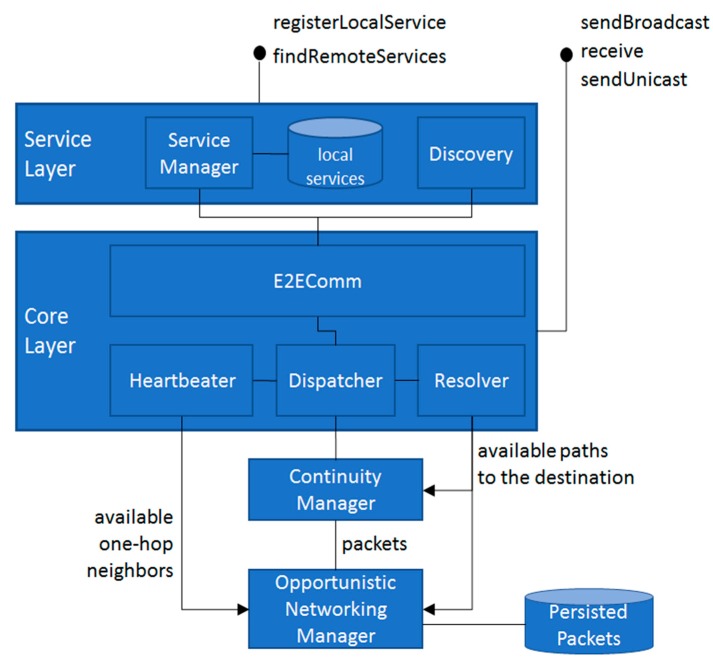
Real Ad-hoc Multi-hop Peer-to peer (RAMP) Opportunistic Networking (RON) architecture.

The RAMP Opportunistic Networking (RON) component (Figure 3) extends the RAMP middleware by enhancing features already provided by the Continuity Manager (CM) component [3]. In particular, CM is in charge of detecting path disconnections and packet dispatching failures (notified by the Dispatcher RAMP component) and trying to take proper countermeasures. CM waits for a short time interval, interacts with the Resolver component in charge of looking for alternative paths to the destination, and sends the packet towards the (possibly discovered) new path. It is worth noting that CM only keeps packets on volatile memory, periodically looks for other paths only for brief intervals (typically in the range of some seconds), and forwards packets only if a path to the destination is found. In cases where a path is not found, the packet is dropped.

RON interacts with CM to intercept packets that would otherwise be dropped, and adopts opportunistic networking techniques [37] to maximize packet delivery probabilities. To this purpose, we have extended the header of RAMP packets by specifying two new metadata:

- an Expiry value in seconds (4 bytes). The sender application exploits novel sendUnicast(…) API to (optionally) specify the Expiry value. Then the RON component exploits this value to understand if and for how long the packet should be managed opportunistically;
- a unique Packet Id (P-ID) that should be valid till the expiration of the packet itself. To this purpose, on the sender side, the RAMP middleware transparently adds a random P-ID (4 bytes) in the header. This solution is envisioned as a proper tradeoff between simplicity of implementation and soundness of the solution, since it is very unlikely that two nodes in the same opportunistic network create the same random packet id, also considering that in most actual scenarios the packet expiry value is typically in the range of hours.

In particular, the primary activities of RON are:

1. RON drops the packet if it is expired (Expire ≤ 0) or if it is a duplicated (by exploiting a volatile database containing the set of P-ID related to already managed packets);
2. in case the packet is still valid, it saves it on persistent memory, eventually removing other persisted packets in case the amount of dedicated memory (configured by the user, mainly dependent on the storage capacity of the node) is full, such as by adopting traditional “remove oldest/biggest” policies (out of the scope of this paper);
3. then, it deserializes packets from the persistent memory in a periodic fashion (typically every 10 s, user configurable) and whenever the CC layer of LCC notifies a network modification. For each deserialized packet, it appropriately decreases the Expiry value, and (if the packet is not expired) exploits the Resolver component to look for a path towards the destination:
  - if a path is found, the packet is sent to the destination and discarded by the local memory;
  - if a path is not found, the packet is duplicated based on the known spray & wait [38] policy (different, more articulated policies can be adopted, but they are out-of-the-scope of this paper) and sent to N one-hop neighbor nodes (N set by the user, default value is 3), with the main purpose of maximizing the probability of correctly dispatching the packet to the destination. To limit networking overhead, nodes take care of not sending the same packet to the same one-hop neighbor more than once. In addition, the destination node should be aware that the same packet could be received more than once, for example, dispatched via different multi-hop paths, taking advantage of the P-ID value to discard duplicated packets.

The source code of the RAMP middleware enhanced with the novel RON component can be found at https://github.com/DSG-UniFE/ramp while the Android porting of RAMP can be found at https://github.com/DSG-UniFE/ramp-android. 

## 6. Quorum-Based Distributed Actuation and Resiliency Policy

As depicted in Section 3, the purpose of the Coordination Wireless IoT Application Layer is to easily coordinate nodes of Wireless IoT Applications by transparently dispatching commands and software components among sensors and controllers. This section addresses the issues raised by the dynamic nature of sparse Smart City environments, preventing controllers from communicating with sensors in a timely manner in case of network partitioning. To this purpose, we propose a quorum-based distributed actuation solution coupled with a resilience behavior policy. In particular, we consider the general case of Internet-enabled controller nodes receiving commands from a cloud-based application and forwarding them to a group of sensor devices to tune their behaviors. Controller nodes are aware of the likeliness that only part of the devices to be controlled could be reachable, while sensor nodes take into consideration the possibility that they may miss commands sent by controllers since the network could be partitioned.

The quorum-based distributed actuation solution defines how controller nodes send control packets to sensor nodes. As a preliminary phase, controller nodes offer a group chat-like service providing a different group for each supported cloud-based application, such as “webcam-based presence detection”. Then, sensor nodes perform a discovery procedure to identify nodes available in the network behaving as controllers for the desired application and select one of the discovered controller nodes supporting the required application and register themselves. The selection is based on QoS parameters, such as path length, round-trip time, or other more sophisticated solutions based on mobility [39,40].

Whenever controller nodes desire to send a command packet to sensor nodes of a given application, they exploit the following protocol:

1. controller nodes use one-to-many semantic to dispatch a pre-command packet to sensor nodes that have previously joined an application;
2. sensor nodes receiving the pre-command packet reply to the controller node with an availability here-I-am packet to confirm their reachability;
3. the controller node, based on the ratio of received/expected availability here-I-am packets and a threshold provided by the cloud-application, determines if the command should be issued or not, sending the actual command packet in the former case.

To improve the resiliency of the network, sensor nodes exploit pre-command packets to be informed that the controller node is still reachable, regardless an actual command packet is then issued or not. In case that pre-command packets are not received for a time period specified by the controller during the join procedure, sensor nodes assume that the controller node is not reachable anymore, e.g., since it has left the network or the network is partitioned. Then, they start again the discovery of controller nodes supporting their applications. In case a controller node is not available, they apply the following resilience policy:

- sensor nodes equipped with Internet-enabled long range wireless technologies (and thus able to behave as controller nodes by interacting with the cloud-based application) start a simple leader-election algorithm (out of the scope of the paper) based on packet flooding to identify a time-limited controller node. Sensor nodes spontaneously participating in the leader-election algorithm are willing to behave as controllers, but only for a brief period of time, to limit power consumption or reduce costs to dispatch the packet to the Internet:
  ○ in case a new controller node becomes available, the sensor node elected as time-limited controller becomes again a sensor node only;
  ○ in case an actual controller is not available for a long time period, the current time-limited controller node stops behaving as controller, to push other Internet-enabled sensor nodes to start the leader-election algorithm again;
- regular sensor nodes (not able to behave as time-limited controller nodes) keep looking for a new controller for a while, that is, to wait for the election of a limited-timed controller node. If a new controller is not discovered, they switch from the “remotely controlled” to the “resilience” state. Whenever in the latter state, they exploit as configuration parameters resilience values (provided via previously received command packets). In fact, since in this case sensor nodes cannot receive command packets from any controller nodes, they cannot discriminate if the active parameter is the same currently set in the cloud application or not. The availability of a resilience parameter issued via controller nodes allows, also in case of network partitioning, to switch to a standard behavior, such as allowing a proper tradeoff between metering frequency and power consumption, also in case of sensor nodes detached from the network.

**Figure 4:**
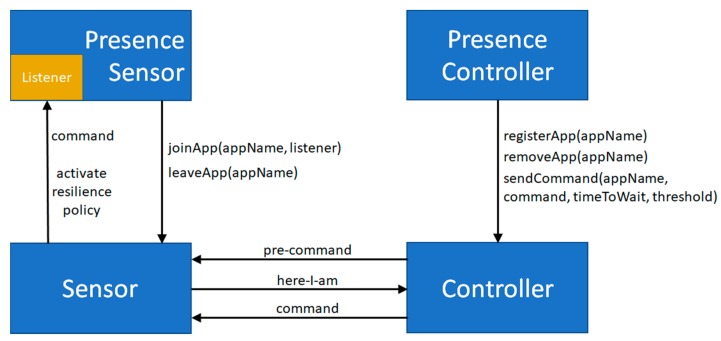
Presence and Controller components.

Figure 4 presents Sensor and Controller components, implementing the quorum-based distributed actuation and resiliency policy solution. The Sensor component offers the following API (Figure 4, left):

- joinApp(appName, listener), allowing to register a listener interested in:
  ○ receiving commands related to a given application;
  ○ being notified of controller unavailability to activate the resilience policy;
- leaveApp(appName), allowing to leave an application and unregister the listener.

The Controller component offers the following API (Figure 4, right):

- registerApp(appName), to add a local application;
- removeApp(appName), to remove a local application;
- sendCommand(appName, command, timeToWait, threshold), to send a command to sensor nodes of a given application: it sends a pre-command, waits for here-I-am messages for timeToWait, and finally sends the actual command message only if at least “threshold” sensor nodes have replied with here-I-am messages.

Pre-command, here-I-am and command messages are dispatched taking advantage of the Multi-hop Networking Overlay Layer, thus transparently achieving multi-hop and opportunistic networking capabilities. As better detailed in Section 8, PresenceSensor and PresenceController components take advantage of the Sensor and Controller ones respectively to transparently register, look for, and join controller nodes, send and receive commands, and notify resilience policy activations in an event-driven fashion. 

## 7. Dynamic Processing Update

New services provided to (and possibly also by) citizens will require the dynamic deployment of new Wireless IoT Applications, by disseminating novel software components or upgrading already deployed ones to take advantage and integrate with additional hardware systems. This observation dictates for a solution enhancing the sparse Smart City scenario by allowing the dynamic distribution, deployment, configuration, exploitation, and upgrade of code interacting with sensors and processing gathered data on nodes composing the network itself. To this purpose, we identify three primary solutions:

1. dynamic code distribution. The behavior specifying how nodes should interact with sensors and process achieved data is distributed to nodes as pre-compiled/script files. In this manner, the amount of code distributed via RAMP packets is minimized, thus allowing to increase the efficiency of behavior change procedures. However, in this case the RAMP middleware is in charge of managing both the distribution and the execution of the code;
2. adoption of the Kura [41] IoT gateway. The adoption of the well-established and maintained open source Kura project can improve the reliability of the solution while minimizing the required maintenance effort. In fact, Kura is a Java-based Open Services Gateway initiative (OSGi) enabled framework already supporting, among the many offered features, to easily manage software components from remote locations;
3. containerization based on Docker [42]. To further support the dynamic configuration and upgrading of nodes, it is also possible to exploit Docker to distribute among nodes not only code, but also the software environment the code should run in. In fact, Docker allows to embed in a single container all the software packages it is required to deploy, thus greatly simplifying the deployment (and upgrade) of new software in heterogeneous devices.

The three solutions above present very different granularity levels; moreover, they present clear pros and cons in terms of standardization, generality of solution, efficiency, and applicability. First of all, Kura and Docker represent open-source solutions gaining momentum in the scientific and industrial community, currently under development and with continuous improvements and upgrades. In terms of generality of the solution, Docker is certainly the best choice, since it can be adopted on top of most spread operating systems, ranging from Windows to many flavors of Linux. In addition, it can contain any type of software environment, thus allowing one to distribute and deploy (almost) any solution. However, Docker containers are usually of relatively large size (likely greater than 100 MB) and thus do not fit strict efficiency requirements in terms of networking traffic of sparse Smart City scenarios, whose nodes are willing to collaborate but could not be available for dispatching huge amount of data. Moreover, Docker cannot be easily deployed on off-the-shelf smartphones, currently requiring rooted Android devices and custom kernels. Kura is already exploited on many devices and its features have been demonstrated to be well-suited on devices with limited hardware resources. However, like Docker, Kura is also not available yet for mobile environments such as Android, thus limiting its applicability. In addition, Kura applies to scenarios where nodes are Internet-enabled and thus it is not suitable for sensors nodes reachable via multi-hop paths.

Based on these considerations, we have opted for a solution based on dynamic code distribution, since it provides larger applicability (it can be easily adopted on any node running the Java-based RAMP middleware) while ensuring limited overhead (it is required to move only the code implementing the new behavior). In the future, we will better investigate the feasibility of the adoption of Docker in sparse Smart City scenarios, eventually exploiting RAMP to dispatch containers while deploying them only on suitable devices, such as Raspberry Pis. 

In particular, we propose a solution based on dispatching and deployment of Java classes and script files, for example, containing Python code. Java classes are distributed as class files and then instantiated interacting with the Class Loader, while scripts are executed as external processes providing their results via the output standard stream or via a support output file. The typical usage scenario consists of a Java class and a script, the former in charge of processing data and the latter of interacting with the sensors. For instance, a Java class periodically activates a Python script, gathers pictures, and compare them to send an alert only if they differ more than a threshold, while the Python script interacts with webcams to take pictures and then provides them via the standard output stream.

The Dynamic Processing Update exploits the one-to-many communication semantic similarly to the quorum-based actuation schema presented in Section 6. Controller nodes behave as software maintainers and suppliers by issuing command packets containing Java classes and/or Python scripts. Sensor nodes interested in receiving up-to-date code related to a specific application specify their interest joining the related group; then, they receive the code on demand, for example, when joining a group, and whenever the controller node distribute an updated version of the software.

## 8. Experimental Results

We have thoroughly tested our prototype to quantitatively evaluate effectiveness and responsiveness along two primary guidelines:

1. evaluation of the RON component while dispatching packets in a sparse Smart City scenario exploiting our LCC and RAMP-WIA. In this case, the main purpose is to quantitatively demonstrate that the proposed solution effectively dispatches packets at multi-hop distance also in a sparse wireless network composed by off-the-shelf devices;
2. evaluation of the quorum-based distributed actuation and resilience policy solution. In this case, the goal is to test the effectiveness of the proposed solution by showing how sensor nodes dynamically discover/join controller nodes and adopt the resilience policy in case a controller node is not available anymore.

In order to estimate the RON component behavior, we have deployed our middleware in a multi-hop spontaneous environment with: Android smartphones with 2.1 GHz Cortex-A53 processor and 3 GB of RAM, running Android 7.0; Raspberry Pi 3 Single Board Computers (SBC) with 1.2 GHz ARM processor and 1 GB of RAM, running Linux Raspbian 8.0; laptops with a 2.70 GHz Intel Core i7-7500U CPU and 16 GB of RAM, running Ubuntu 17.04 Zesty Zapus; laptops with a 2.70 GHz Intel Core i7 CPU and 12 GB of RAM, running Ubuntu 16.04.02 Xenial Xerus. Let us stress that the main goal of this section is to verify and assess the effectiveness of the proposed approach by focusing on a few key aspects of the implemented RAMP-WIA middleware. In particular, performance results reported below are based on experimentation over real and off-the-shelf devices, with the main purpose of testing the technical soundness and practical feasibility of the communication and coordination mechanisms used in RAMP-WIA. More extensive and comprehensive performance results, based on a city-wide large-scale testbed, are out of the scope of this paper and one of the most relevant foci of our future research work.

**Figure 5:**
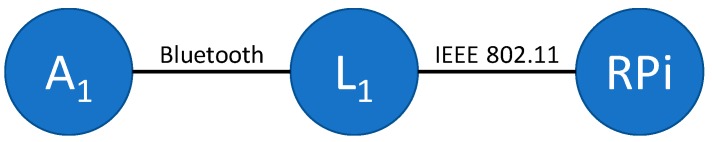
Two-hop deployment scenario.

First of all, we have tested the two-hop environment in Figure 5, formed by a Bluetooth hop between an android device (A_1_) and a laptop (L_1_) and a IEEE 802.11 hop between L_1_ and a Raspberry Pi 3 (RPi), performing the following steps:

- t_0_: A_1_ sends a packet to RPi but the Bluetooth is not available and the packet is persisted by RON;
- t_1_: A_1_ (manual configuration) creates a Bluetooth link with L_1_;
- t_2_: after a while RON on A_1_ retrieves the persisted packet and looks for the destination node RPi;
- t_3_: RON on A_1_ does not find any available path towards RPi since the IEEE 802.11 link is not available. Therefore, it sends the packet to the neighbor node L_1_;
- t_4_: L_1_ receives the packet and tries to dispatch it to RPi. However, since there is no path currently available, RON on L_1_ persists the packet;
- t_5_: L_1_ creates a IEEE 802.11 link with RPi (manual configuration);
- t_6_: after a while RON on L_1_ retrieves the persisted packet and looks for the destination node RPi;
- t_7_: RON on L_1_ finds a path towards RPi via the IEEE 802.11 link and thus it sends the packet to RPi.

We have tested the sequence above 10 times, achieving an average end-to-end packet dispatching time of 115.8 s with a standard deviation of 4.8, demonstrating that the proposed solution can dispatch packets towards the destination in environments characterized by frequent network partitions, by fully exploiting any connectivity opportunity. Note that the previous values also include the time required to manually configure the network, which comprises the time between t_0_ and t_1_ and the time between t_4_ and t_5_ estimated in 55 s per-hop. In other words, measured values include, on the one hand, the time to wait for manual network configuration and, on the other hand, the time required for RON to periodically retry packet transmissions (period of 10 s). However, it takes only 5.8 s (115.8 s–55 s × 2) to dispatch packets if not considering the time required to manually reconfigure the network. In particular, when a node activates a new link with another RAMP node, it spends about 2.9 s to forward packets due to RAMP neighbor discovery and RON procedures (comprising periodic waiting time, packet restoring, and packet forwarding to new neighbors).

**Figure 6:**
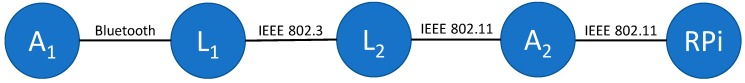
Four-hop deployment scenario.

Then, we have extended the previous scenario with additional devices between A_1_ (the sender) and RPi (the receiver) and by making LCC work by automation with lazy and hectic policies (see Figure 6). This scenario also includes an always-on Ethernet link, while IEEE 802.11 and Bluetooth links are periodically activated and deactivated by LCC (at startup wireless links are not available). Furthermore, LCC on node A_2_ is in client-only role, switching among the two IEEE 802.11 networks towards L_2_ and RPi. 

When adopting lazy and hectic policies, the end-to-end packet dispatching takes on average 365.4 s and 158.9 s respectively. Therefore, it is possible to see that the hectic policy relevantly lowers the required time thanks to reduced HC, RS, and RW values. However, the required time is still relatively high due to two main issues. First of all, when the Bluetooth link between A_1_ and L_2_ is used, LCC requires about 93 s to actually activate IP connectivity (considering Bluetooth connection and DHCP protocol) while RON takes about 10 s to become aware that there is no path towards RPi (7.5 s to retry 3 times to send the packet towards the previously known path and 2.5 s to unsuccessfully look for an alternative path). Secondly, when node A_2_ receives the packet from L_2_, it has just switched network and then it has to wait for the following hotspot change period. It is worth noting that whenever LCC modifies network configurations it sends a message to the local RON, thus activating the latter as soon as additional connectivity opportunities arise and improving the efficiency of packet delivery. Considering only RON tasks and packet transmission, the delay of the first three hops is about 2.7 s per hop, of which 2.5 s to look for RPi (always unsuccessfully since there is no multi-hop path towards RPi) and the rest to transmit the packet to the newly discovered one-hop neighbor (since RPi is not available). Instead, in the last hop A_2_ successfully looks for RPi (the final destination of the packet) and sends the packet in about 0.2 s.

**Figure 7:**
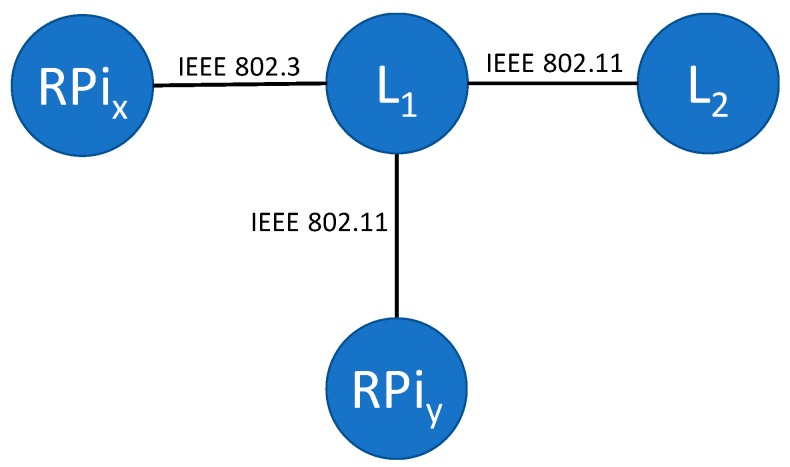
PresenceSensor application testing deployment.

To evaluate the quorum-based distributed actuation and resilience policy solution we have developed a PresenceSensor Java application (based on the Sensor component) running on two webcam-enabled Raspberry Pis and a laptop (nodes RPi_x_, RPi_y_, and L_2_, Figure 7) periodically acquiring images webcam and sending alert messages only if the previous and current images differ more than a threshold. In addition, a PresenceController application (based on the Controller component) runs on a laptop (node L_1_, Figure 7). Delving into finer details:

- PresenceController on node L_1_ registers itself as a controller node offering the “presence” application;
- PresenceSensors on nodes RPi_x_ and RPi_y_ look for a controller node offering the “presence” application, and then join it;
- PresenceSensors on nodes RPi_x_, RPi_y_, and L_2_ periodically take pictures and broadcast alert messages whenever images differ more than 40% (default value);
- PresenceController on node L_1_ periodically sends pre-command messages to sensor nodes in the “presence” group;
- PresenceSensors on nodes RPi_x_, RPi_y_, and L_2_ reply with an here-I-am message to node A.

After a while, the PresenceController sends a command to sensor nodes of the group “presence” with following values:

- sensor node rate: 100%, that is., the command message is sent only if every sensor node is available;
- current image difference value: 20%, that is, alert messages are sent if images differ more than 20%;
- resilience image difference value: 50%, that is, in case of controller unavailability, alert messages are sent whenever images differ more than 50%.

To this purpose, the PresenceController on node L_1_ exploits Controller to send a “pre-command” message to sensor nodes:

- if every sensor node replies with a “here-I-am message” (default wait timeout of 3 s), node L_1_ actually sends the command. When sensor nodes receive the command, Sensor components exploit the registered listener to forward the command to PresenceSensor instances;
- otherwise, controller node L_1_ does not send the command, since the sensor node rate requirement is not fulfilled.

We have tested the above scenario in two cases: (i) the three sensor nodes are available; and (ii) only RPi_x_ is available. In the former case, after 900 ms on average, the controller node receives the here-I-am messages and sends back the actual command message. PresenceSensor components on sensor nodes receive the command and start sending alert images with image threshold at 20%. In the latter case, the controller node receives the here-I-am message only from node RPi_x_ and, after a waiting time of 2 s (configurable parameter), aborts the command procedure.

Then, we have removed the controller node L_1_ from the network. After 5 s, Sensor components on sensor nodes perceive that there is not a controller node any more (since they do not receive periodic pre-command messages) and start a discovery procedure to look for a new one (since the three nodes are not Internet-enabled, they do not start the leader-election algorithm). After 20 s (configurable parameter), the Sensor components realize that there is no controller in the network and thus exploit the listener-based architecture to send to PresenceSensor instances an “activate resilience policy” event.

## 9. Conclusions

The paper presents a novel solution supporting the development and dynamic deployment of applications in infrastructure-less wireless environments, specifically considering sparse Smart City environments where mobile devices carried by users collaborate to support the remote monitoring and control of sensor nodes. In particular, the proposed RAMP-WIA middleware allows dynamic management of mobile nodes, not only at the communication level, by creating new single-hop links and opportunistically dispatching packets at multi-hop distance, but also at the application level, by allowing software components on mobile nodes to be deployed and upgraded on-the-fly. The RAMP-WIA middleware also takes into consideration that the greatly dynamic nature of sparse Smart City scenarios may not allow to dispatch commands to sensor nodes in a timely manner. For this reason, it dispatches commands only if a quota of sensor nodes is currently reachable and supports a resiliency policy that allows sensor nodes detached from the network to properly reconfigure themselves while waiting to restore connectivity with a controller.

The encouraging results achieved based on the working implementation of the RAMP-WIA middleware are stimulating our ongoing research work. We are mainly working on the containerization of the implemented middleware as well as of specific sensor/controller components. In addition, we are investigating the adoption of Software Defined Networking (SDN) based approaches to improve the QoS by appropriately managing different traffic flows, by avoiding interferences, and by considering network capabilities/conditions combined with application-level requirements. Finally, we intend to perform wider and more in-depth performance analyses based on a real-world testbed with the scale expected for the targeted smart city execution environments, that is, including thousands of off-the-shelf devices.
